# A comparative analysis of DNA methylation across human embryonic stem cell lines

**DOI:** 10.1186/gb-2011-12-7-r62

**Published:** 2011-07-06

**Authors:** Pao-Yang Chen, Suhua Feng, Jong Wha Joanne Joo, Steve E Jacobsen, Matteo Pellegrini

**Affiliations:** 1 Department of Molecular, Cell, and Developmental Biology, University of California, Los Angeles, CA 90095, USA; 2 Institute of Genomics and Proteomics, University of California, Los Angeles, CA 90095, USA; 3 Interdepartmental Program in Bioinformatics, University of California Los Angeles, Los Angeles, CA 90095, USA; 4 Howard Hughes Medical Institute, University of California, Los Angeles, Los Angeles, CA 90095, USA; 5 Broad Center of Regenerative Medicine and Stem Cell Research, University of California, Los Angeles, Los Angeles, CA 90095, USA; 6 Molecular Biology Institute, University of California, Los Angeles, CA 90095, USA

## Abstract

**Background:**

We performed a comparative analysis of the genome-wide DNA methylation profiles from three human embryonic stem cell (HESC) lines. It had previously been shown that HESC lines had significantly higher non-CG methylation than differentiated cells, and we therefore asked whether these sites were conserved across cell lines.

**Results:**

We find that heavily methylated non-CG sites are strongly conserved, especially when found within the motif TACAG. They are enriched in splice sites and are more methylated than other non-CG sites in genes. We next studied the relationship between allele-specific expression and allele-specific methylation. By combining bisulfite sequencing and whole transcriptome shotgun sequencing (RNA-seq) data we identified 1,020 genes that show allele-specific expression, and 14% of CG sites genome-wide have allele-specific methylation. Finally, we asked whether the methylation state of transcription factor binding sites affects the binding of transcription factors. We identified variations in methylation levels at binding sites and found that for several transcription factors the correlation between the methylation at binding sites and gene expression is generally stronger than in the neighboring sequences.

**Conclusions:**

These results suggest a possible but as yet unknown functional role for the highly methylated conserved non-CG sites in the regulation of HESCs. We also identified a novel set of genes that are likely transcriptionally regulated by methylation in an allele-specific manner. The analysis of transcription factor binding sites suggests that the methylation state of *cis*-regulatory elements impacts the ability of factors to bind and regulate transcription.

## Background

Epigenetic regulation, such as cytosine DNA methylation, is important in gene regulation. Inappropriate methylation and silencing of tumor suppressor genes, and the inappropriate loss of DNA methylation of oncogenes, have been recognized in recent years as key factors in the development of cancer [1]. DNA methylation changes are also critical in the differentiation of cells, as seen for example in embryonic stem cells (ESCs) [2].

It is possible that DNA methylation mediates these effects by altering interactions between transcription factors (TFs) and DNA. TFs bind to specific sequences on DNA (that is, TF binding sites (TFBSs)) to initiate transcription [3]. DNA methylation may regulate transcriptional programs by directly impacting the binding of TFs to DNA, although to date there is little direct evidence of this. However, it is thought that promoter CpG islands are generally unmethylated to facilitate DNA binding with transcription factors [4], and changes of methylation at promoter CpG islands can directly influence gene expression levels. It has also been shown that several *cis*-regulatory elements can directly influence the methylation of CpG islands within the promoter regions [5,6]. Nonetheless, genome-wide relationships between TF activities and the methylation state of *cis*-regulatory elements have to date not been convincingly established.

One aspect of DNA methylation-induced transcriptional regulation that has been extensively studied is allele-specific transcription from either the maternal or paternal chromosomes [7]. Some of these allele-specific events may be regulated by DNA methylation though mechanisms such as imprinting [8], inactivation of × chromosomes [9], or non-imprinted allele-specific methylation [10]. Imprinting leads to the expression of only the paternal or maternal allele, depending on the locus. A recent study on the mouse brain reported that more than 1,300 loci are affected by the parent-of-origin allelic effect [11] and are candidates for imprinted genes. In addition, it has also been reported that about 10% of all human genes are regulated by non-imprinted allele-specific methylation [10]. The allele-specific methylation of these genes is associated with genetic polymorphisms and may also correlate with allele-specific expression. Other allelically imbalanced genes have been shown to have random mono-allelic expression [12]. It is estimated that one-third of these genes with random mono-allelic expression are determined by alleles rather than parent of origin and are likely to be regulated by *cis*-acting factors [13,14]. Nonetheless, to date it has not been possible to simultaneously study allele-specific methylation and transcription in a single sample, and therefore the degree to which these are related is still not known.

DNA methylation-driven transcriptional regulation is known to play a significant role in the establishment of cellular differentiation programs. To investigate the role of DNA methylation in these cellular programs, several studies have reported the comparisons of methylation profiles between ESCs (or multipotent progenitors) and differentiated cells [15-18] and induced pluripotent stem cells [19,20]. These vertical comparisons provide valuable insights into the dynamic changes of methylation in development. For example, they reported that non-CG methylation is present at low levels in human ESCs (HESCs), but disappears upon induction of differentiation of the ESCs, and is restored in induced pluripotent stem cells [15], suggesting there may be a functional role for non-CG methylation in pluripotent stem cells.

However, less is known about the conservation and variability of DNA methylation across different stem cell lines. A recent analysis of about 1% of the genome of HESC lines shows that, by monitoring DNA methylation and gene expression, it is possible to identify cell line-specific defects that could interfere with their differentiation or the functional properties of derived cell types [19]. Using genome-wide bisulfite sequencing (BS-seq) [21], we have recently determined the DNA methylation profile of the human embryonic stem cell line HSF1 [22]. BS-seq is able to generate genome-wide DNA methylation profiles at single base resolution, much improved from previous profiling methods limited by low resolution [23,24] or sequence-specific biases [25]. Here we report a comparison of the methylation profile of HSF1 with those from two other HESC lines: H1 [15] and H9 [16] (also known as WA09). We are for the first time able to address questions about the conservation of methylation at non-CG sites across HESC lines. Furthermore, we have developed a novel approach to measure allele-specific expression by combining BS-seq and RNA-seq data from the same sample. RNA-seq provides digital measurement of transcription at single base resolution, and thus allows us to perform genome-wide scans for mono-allelically expressed genes by associating exonic SNPs (detected from BS-seq data) with their allelic expression levels (from RNA-seq). From BS-seq data we also identified CG sites that are differentially methylated between the two chromosomes, resulting in allele-specific methylation. Hence, we can identify genes with allele-specific expression and methylation. Using our methodology, we found that one-third of the genes have allele-specific expression, and identified a set of differentially methylated genes that are enriched for allele-specific expression. Finally, we measured the methylation levels at TFBSs throughout the genome and correlated them with gene expression levels. We were able to compare the methylation levels at the same binding site across all three cell lines. We identified several factors that show significant correlation that are even more correlated at the binding sites than the neighboring sequences, suggesting for the first time that their binding affinities are directly regulated by the methylation of *cis*-regulatory elements

## Results

We aligned bisulfite converted reads from the HSF1, H1 and H9 cell lines using BS Seeker [26] to reduce any mapping bias that might have been caused by different mapping approaches used in the original publications (see Materials and methods). We mapped 684 million, 763 million and 792 million reads to unique positions in the genome for HSF1, H1 and H9 with an average coverage of 10x, 20x, and 16x, respectively (Table S1 in Additional file 1). Methylation levels at each cytosine were determined by measuring the ratio of Cs to Cs plus Ts that align to each genomic cytosine. The data can be browsed through at [27].

### Global methylation differences

We compared global methylation levels between the three cell lines. We estimate average methylation levels across the genome (that is, the chance that a cytosine is methylated) by computing the mean value of the number of methylated reads over the total number of reads mapped to each cytosine. For these estimates we consider only cytosines that are covered by at least four reads. As expected, most CG sites are highly methylated (see Table 1 for global methylation levels). From the histogram of methylation levels (Figure S1 in Additional file 1), we observe a bimodal distribution of methylation, which indicates a significant part of CG sites are weakly methylated. In contrast, non-CG sites are generally not methylated or weakly methylated, although their methylation levels vary depending on the adjacent nucleotides. Interestingly, we observe significant differences in the global methylation levels between cell lines; the CG methylation level is highest in H1 at 85%, followed by HSF1 at 75%, and lowest in H9 at 72%. A similar trend is also observed for non-CG methylation. The differences in methylation levels may be due to a combination of effects, such as the unstable dynamic gain and loss of methylation reported in ESCs [28,29], and protocol- and lab-specific differences between the data sets (for example, passage number in Table S1 in Additional file 1).

**Table 1:** Methylation levels (percentage) of H1, HSF1 and H9 cell lines in various genome contexts

HESC line	CG	CHG	CHH	CA	CT	CC	CAG	TACAG
H1	84.70	3.62	1.48	3.56	1.09	0.67	5.84	21.87
HSF1	74.96	2.99	1.39	2.76	1.14	0.93	4.38	12.96

H9 (WA09)	71.74	1.76	0.73	2.02	0.55	0.26	3.02	14.13

H = A, C, or T. In CC context, the reported values is based on the first C.

We performed a genome-wide screen for regions that are differentially methylated between pairs of cell lines, and identified between 1.4 and 2% of the genome that is significantly differentially methylated at CG sites. Of these regions, 6% are overlapping between the three cell lines (false discovery rate (FDR) = 0.5%; see Materials and methods). These overlapping differentially methylated regions are enriched in promoters, exons, and most significantly in CpG islands (Figure S2a in Additional file 1). The overlapping differentially methylated CHG (where H is A, T or C) regions are most enriched in exons, and CpG islands (Figure S2b in Additional file 1). This result contrasts with previous reports that concluded that CpG islands did not have significant methylation variability across samples, which was primarily constrained to the shores of the islands [30]. Both promoter CG methylation and non-CG methylation within genes have been reported to correlate with gene expression [6,15]. Thus, the enrichment of differential methylation in these regions may influence transcriptional rates, although a direct causal connection cannot be established with our data. Figure S3a in Additional file 1 shows that, as expected, significantly differentially methylated CpG islands are negatively correlated with gene expression (see Additional file 2 for lists of associated genes). The correlation in CpG island shores is, however, less clear (Figure S3b in Additional file 1). An analysis of the gene ontology terms for genes associated with these differentially methylated CpG islands shows that their functions are enriched for transcription regulation, neuron differentiation, and genetic imprinting (via David bioinformatics resources [31]).

### Lowly methylated CG sites are conserved

The recent analysis of methylomes has shown that unlike differentiated cells, HESC lines have significant levels of non-CG methylation that account for up to 25% of all methylated cytosines. Whether these methylated non-CG sites are conserved across different lines was not previously known. We computed the conservation of methylation by carrying out pairwise comparisons of the three cell lines at single base resolution. The conserved and unconserved sites are defined as those that have either concordant or discordant methylation levels between the cell lines. Cytosines were categorized into three groups according to their methylation levels. For CG sites, the grouping is low methylation (0 to 33%), median methylation (34 to 66%), and high methylation (67 to 100%), while for non-CG sites the groups are no methylation (0%), low methylation (0 to 30%), and high methylation (31 to 100%). The cutoff values for CG methylation are higher than non-CG because CG sites are significantly more methylated than non-CG sites, and their distributions of methylation levels are bimodal. The methylation at a cytosine site is considered conserved if this cytosine is categorized into the same group in both cell lines; otherwise it is unconserved.

**Figure 1:**
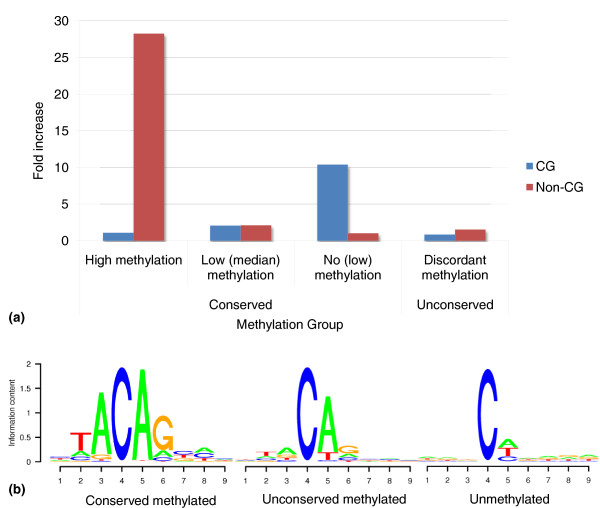
**Conservation and DNA methylation of CG and non-CG sites**. **(a) **Fold enrichment of CG and non-CG sites grouped by their methylation and conservation. **(b) **Sequence motifs for 'conserved highly methylated', 'unconserved methylated' and 'unmethylated' non-CG sites. The motifs show the averaged result from the pairwise comparisons between the three cell lines.

The number of cytosines in the groups is compared to a null model that assumes the independence of methylation between the two cell lines. Thus, the more significant the deviation between the observed data and the null model, the more significant the conservation of methylation between the two cell lines. Figure 1a shows a summary of the results from the three pairwise comparisons (see Figure S4 in Additional file 1 for the pairwise comparisons). We find that lowly methylated CG sites and highly methylated non-CG sites are strongly conserved. On average, 6% of the CG sites are conserved in a low methylation state in pairwise comparisons of cell lines. These conserved sites are enriched in promoter regions (Figure S5 in Additional file 1) and CpG islands, which are generally demethylated.

### TACAG sites are conserved and highly methylated

In contrast to CG sites, we find that only the highly methylated non-CG sites are conserved across the three ESC lines, while the poorly and non-methylated sites are not. Overall, conserved highly methylated non-CG sites are rare (only 0.2% of all non-CG sites) and are enriched in genes (Figure S6 in Additional file 1).

We performed an analysis of the sequence motifs associated with non-CG sites that are conserved highly methylated, unconserved methylated, and unmethylated. The unconserved methylated sites are those highly methylated in one cell line and unmethylated in the others. We found the motif TACAG is enriched in conserved highly methylated non-CG sites, whereas unconserved but generally methylated sites are enriched for CA (or less strongly CT) (Figure 1b). Lister *et al*. [15] have previously reported that the TACAG motif is enriched for methylation. Here we further establish that the 'TA' dinucleotide sitting immediately upstream of 'CAG' is typically observed with conserved methylation, suggesting a strong methylation preference holds across human ESC lines. The methylation level of TACAG sites is 22%, which is strikingly higher than other non-CG contexts (for example, CHG is 3.6%, CA is 3.6% and CAG is 5.8%).

**Figure 2:**
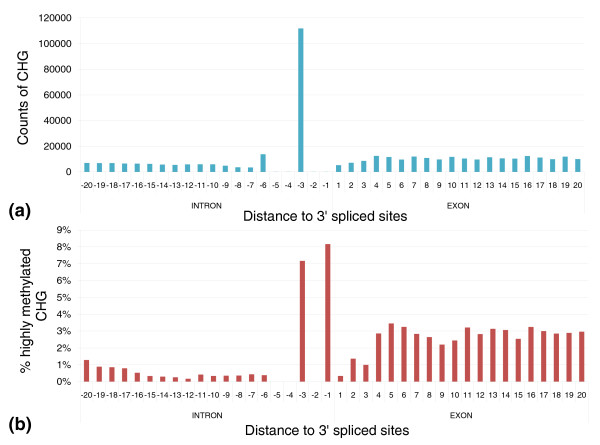
**Distribution of CHG sites at 3' splice sites and their methylation levels**. **(a) **Counts of CHG sites. **(b) **Percentage of highly methylated CHG in 3' splice sites.

**Figure 3:**
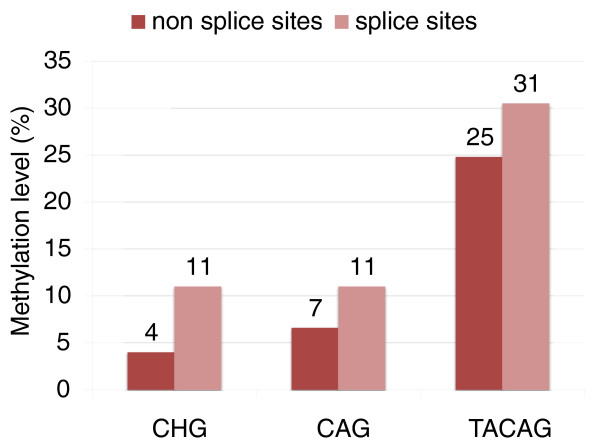
**Methylation levels of non-CG sites within the gene body in splice sites and non-splice sites**.

The TACAG motif is methylated at a cytosine that we refer to as CHG (where H is A, T or C). CHG sites are generally enriched in exons, and frequently observed at splice sites. The methylation of CHGs is slightly higher in exons than in introns (Figure S7 in Additional file 1). At the third position upstream of the 3' splice site where the sequence CHG is highly enriched (due to the presence of the canonical acceptor sequences), we observe high levels of methylation (Figure 2; Figure S8 in Additional file 1). More than 99% of the cytosines at this position are in CAG sites, and 8% are in TACAG motifs. Since CAG and TACAG sites are much more methylated than all CHG sites, the methylation level at this position is higher than the average found in introns and the entire genome. A similar trend is also observed at 5' splice sites (Figure S9 in Additional file 1). Since CHG methylation is usually enriched in genes [15], we found that CHG in splice sites is even more methylated than other CHG sites within genes (Figure 3). While the mechanistic connection between DNA methylation and splicing is still not clear, Laurent *et al*. [16] also reported high levels of CG methylation at the 3' splice sites. Furthermore, we found that, in all cell lines, alternatively spliced exons have lower CG and non-CG methylation compared to interior exons (Figure S10 in Additional file 1), suggesting that a relationship may exist between methylation of exons and alternative splicing.

### Symmetry of CG and non-CG methylation

In mammals, DNA methylation is established by the *de novo *methyltransferase DNMT3 [32-34] during early embryogenesis. The maintenance methyltransferase DNMT1 methylates hemi-methylated CG sites during DNA replication, leading to symmetrically methylated CG sites [15,35]. Whether there is any mechanism for recognizing hemi-methylated CHG sites and methylating the other strand is still not known. To assess the symmetry of methylation at CG and CHG sites, we analyzed two-by-two contingency tables containing the methylation status of C and G (that is, C on the antisense strand) as the two factors. Confirming previous analyses [15], we found that more than 77% of CG sites are symmetrically methylated on both strands, whereas only about 0.2% of CHG sites are symmetrically methylated. The observed counts in the table are compared against the expected values based on the assumption that methylation at C and G is independent. Interestingly, we found that, in all cell lines, the methylation in lowly methylated CG sites (that is, < 30%) is much more symmetric (Figure S11 in Additional file 1) than expected, which may be associated with the symmetric demethylation found within CpG islands [4]. On the other hand, we found that the symmetric methylation at highly methylated CHG sites (that is, > 30%) is observed significantly more than expected (Figure S12 in Additional file 1). The symmetry of methylation in lowly methylated CG and highly methylated CHG sites is consistent with the observation that both these types of sites are conserved across cell lines.

### Allele-specific expression

We developed a novel methodology to study the relationship between allele-specific transcription and methylation on a genome-wide scale. To accomplish this, we integrated the BS-seq data with RNA-seq data to first perform a genome-wide scan for genes with allele-specific expression. Using BS-seq data from the H1 cell line, we searched for genes that contain SNPs located within transcribed regions (exonic SNPs; see Materials and methods for details). Since bisulfite converted DNA creates ambiguities between cytosines and thymines, we discarded reads with Cs and Ts that mapped to Cs on either strand of the genome. The two alleles in an exonic SNP arise from differences between the two parental alleles. The allele to which the majority of RNA-seq reads map (from H1 RNA-seq data) is considered the major allele and the other the minor allele (that is, highly expressed and lowly expressed allele). Genes with allele-specific expression may have significantly uneven numbers of RNA-seq reads aligning to major and minor alleles. In our dataset, we found 7,109 exonic SNPs covering 3,704 genes. To be called a SNP, a locus had to have a coverage of at least eight reads, and a ratio between 0.5 and 0.6 for the major allele. For each gene we calculated the probability that the major and minor alleles are unbalanced based on a binomial test computed from the number of reads covering the major and minor alleles. For this test the null hypothesis is that two alleles are equally covered and genes with *P*-values < 0.0027 (corresponding to a 1% FDR) are deemed mono-allelically expressed. In total, we identified 1,020 genes with allele-specific expression, or 28% of the total genes with at least one exonic SNP. The full list of these genes with allele-specific expression is available in Table S3 in Additional file 3.

The number of our predicted genes with allele-specific expression is close to the number (1,306 loci) reported in a recent genome-wide survey in mouse [11]. The percentage of our genes is close to the previously reported value of 28% that were shown to have strong signals for allelic imbalance in other studies [36]. Figure S13 in Additional file 1 shows that, in general, the genes with allele-specific expression have higher gene expression levels than the genes without.

We obtained a list of 75 imprinted genes from the literature [37,38] that we expect to show allele-specific expression (see Additional file 4 for a list of imprinted genes). Of these, 14 were covered by our SNPs and could therefore be analyzed using our binomial test. We observed significant *P*-value scores for 7 of the 14 imprinted genes, confirming that the known imprinted genes are enriched for allele-specific expression (*P *= 0.018, hypergeometric test). The other seven imprinted genes failed to show significant enrichment in our list due to low SNP coverage (only one or two SNPs), which limits the power of our test.

### Allele-specific methylation

We next searched for genes that are methylated in an allele-specific manner, and asked whether these genes are associated with allele-specific expression. From our analysis we do not know the paternal and maternal genotypes, but can identify cytosines that are differentially methylated between two parents, that is, the methylation status may be high in the paternal chromosomes and low in the maternal one (or vice versa). From the SNPs we are able to assign reads to one of the two alleles. The cytosines covered by these reads can be tested for differential methylation. A candidate cytosine is considered differentially methylated if the methylation levels between the reads from the two parents are significantly different (see Materials and methods). Overall, we found that 14% of the candidate cytosines are differentially methylated (these sites are available through the genome browser at [27]). Differentially methylated promoter sites are difficult to detect because CG sites are generally demethylated and also promoter regions are small. We searched for genes enriched with differentially methylated sites in three cell lines. As a result, we found 110 genes are significantly enriched with differentially methylated cytosines in at least one cell line (see Additional file 5 for the gene list). Of these, ten were found in multiple cell lines and eight of these have at least one exonic SNP and could be tested for allele-specific expression. Strikingly, we found that six of the eight genes with allele-specific methylation also show allele-specific transcription. We hypothesize that the allele-specific expression of these genes is regulated by DNA methylation, and that these genes may represent previously unknown imprinted genes. While most genes with allele-specific expression are not enriched with allele-specific methylation, many of them may still be transcriptionally regulated by a single site with allele-specific methylation.

In order to better understand the distribution of differentially methylated CG sites and its relationship with allele-specific expression, we reconstructed the methylation status for the major and minor alleles of all genes. We tested whether the segregation of the major and minor alleles in the exonic SNPs results in two distinct methylation patterns on each chromosome, one of which is highly methylated and the other one unmethylated (or weakly methylated). We were able to associate methylation patterns at the CG sites with major and minor alleles if the SNPs and the CG sites are spanned by the same read (see Materials and methods).

**Figure 4:**
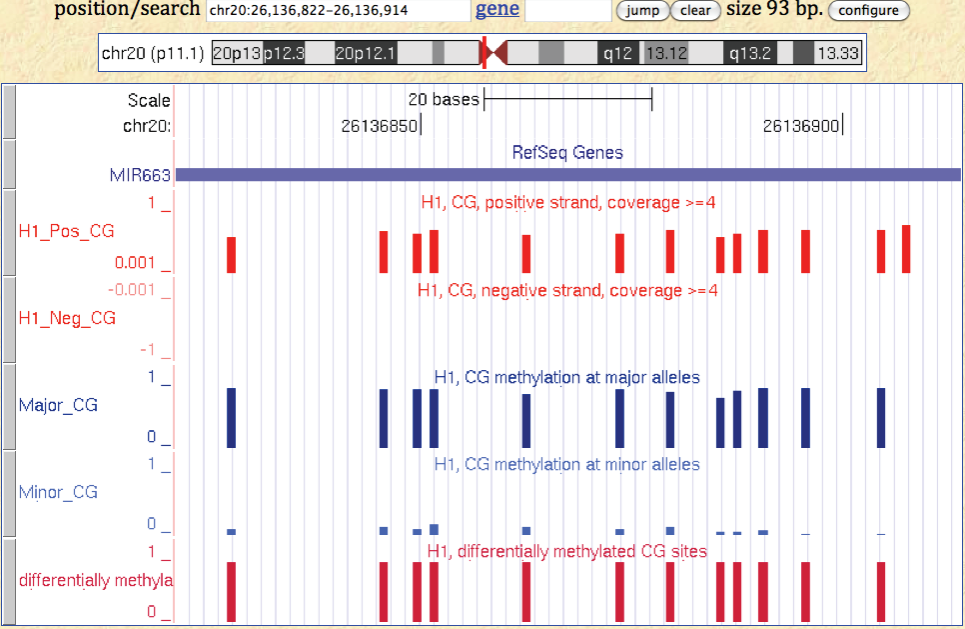
**Distinct methylation patterns between the two reconstructed parental sequences of *mir663***. Differentially methylated CG sites are found within *mir663*. BS-seq mapping shows intermediate methylation levels. The reconstruction of two parental chromosomes reveals that methylated cytosines are associated with expressed alleles.

We expect for genes showing both allele-specific methylation and expression, the major forms arise from one parental chromosome, and the minor from the other. *mir663 *(HUGO Gene Nomenclature Committee (HGNC) ID [HGNC:MIR663]) is found to have a cluster of 12 differentially methylated CG sites located within its gene body of 93 bp. Although with only one exonic SNP, *mir663 *is not significant in our test of allele-specific expression. It has distinct methylation patterns between the two parental chromosomes that can be associated with allele-specific expression (Figure 4), suggesting one chromosome is fully methylated while the other fully unmethylated. However, for most genes this bimodal trend of methylation patterns is only observed in local regions spanning a few CG sites in the gene body, suggesting the effects of allele-specific methylation may appear only at specific sites instead of spanning throughout the gene body.

### Differential DNA methylation in transcription factor binding sites

It has been previously reported that TFBSs tend to be de-methylated [4,6,15] in order not to destabilize the interaction between DNA binding proteins and their target sequences. However, we observed a high variance of methylation at TFBSs (Figure S14 in Additional file 1), suggesting that methylation does occur in some sites. To determine the effects of the methylation of *cis*-regulatory binding motifs on transcriptional regulation, we compared the changes of methylation levels between pairs of cell lines at binding sites with the changes of the expression levels of their associated genes.

The coordinates of TFBSs were downloaded from Motifmap [39] (sites with FDR < 0.1). We determined the methylation level of these sites in the three cell lines, and associated each site with its corresponding gene expression data (obtained from the Gene Expression Omnibus database [GSE9448]). We were able to include 14,000 to 25,000 TFBSs from 125 to 164 motifs (45 to 64 TFs, varied by pairwise comparisons of cell lines). For each motif associated with a TF, we calculated the global correlation coefficient between the change in methylation and the change in gene expression over all the TFBSs where differential methylation was observed (see Additional file 6 for a list of TFs, the methylation level at the motifs and at the neighboring sequences, and the correlation coefficients). If we observed a significant correlation, we hypothesized that the DNA methylation state of the binding site affects the function of the associated TF. Furthermore, we compared the correlation with that in neighboring sequences, defined as ± 500 bp around the binding sites, to assess whether the factor is being affected by specific changes in methylation of the binding site, instead of more general methylation changes in the surrounding region. In these comparisons we matched the two cell lines being compared, the genomic context, and the motif, and restricted the analysis to those that had at least ten binding sites and a *P*-value of the Pearson correlation coefficient less than 0.05. We identified 22 motifs that satisfy these criteria, 17 of which show higher correlation with gene expression than neighboring sequences. We conclude that, for these motifs, the binding of the associated TFs depends on the methylation state of the cytosine(s). To our knowledge, this is the first systematic demonstration that TF-DNA interactions are sensitive to cytosine methylation.

Among the DNA methylation sensitive motifs we identified SP1 [HGNC:SP1], which regulates the expression of genes involved in a variety of processes, such as cell growth [40], apoptosis [41], and embryonic development [42]. The motif M00932 in SP1 shows greater anti-correlation than the neighboring sequences, which suggests a specific association with the methylation of the binding sites. Other TFs we identified, such as RP58 (aka [HGNC:ZNF238]), yielded a positive correlation between methylation changes and expression levels (that is, greater methylation on the motif increased expression levels). RP58, a transcriptional repressor found at transcriptionally silent heterochromatin, associates with DNMT3A, independently of its *de novo *methylation activity, to repress transcription [43,44]. The methylation level at the motif M00532 in RP58 is also more correlated with expression than the neighboring sequences. Two motifs showed opposite correlation trends with their neighboring sequences: CREB (cAMP response element-binding) [HGNC:CREB] and MEIS1A (isoform of [HGNC:MEIS1]). The CREB binding sites are positively correlated with expression whereas the neighboring sequences are anti-correlated. The positive correlation may be due to the fact that CREB is known to be able to repress transcriptional activity [45]. MEIS1A binding sites are anti-correlated with expression whereas its neighboring sequences are positively correlated. The MEIS1A carboxyl terminus harbors a transcriptional activation domain that is stimulated by protein kinase A in a manner dependent on the co-activator of CREB [46]. So it is possible that the methylation status at their binding sites is associated with the binding of CREB and MEIS1A that jointly affect the expression of associated genes.

## Discussion

### Global methylation levels

We performed a comprehensive comparison of the methylation patterns in three human ESC lines to explore their differences as well as their similarities. We found that their absolute methylation levels are different. The reason for this may be due to a number of factors, including different library preparation techniques used in the three different studies, variabilities between sequencing runs, or *bona fide *biological differences between the methylation levels of the three cell lines. We suspect that the 13% difference between these lines is greater than the variation in global methylation found across biological replicates and different runs, which is typically significantly smaller. It is also shown in a recent study that cell passage-related 'biological variation' in methylation is present but minimal on the scale of the genome [47]. We therefore hypothesize that these differences represent true variation in global methylation levels between the three lines. However, until a systematic study of all three lines is performed by a single lab using identical protocols for all three lines, it may be difficult to determine the relative influence of these factors. Nonetheless, it is interesting to note that there are known phenotypic differences between the three lines that could potentially be due to variabilities in their global DNA methylation levels. It has been demonstrated that some HESC lines have a propensity to differentiate into specific lineages [19]. For example, HUE 8 more efficiently differentiates into pancreatic cells than other lines [48], and H1 yields robust hematopoietic lineages whereas HSF1 does not (unpublished data). Furthermore, it has been reported that the differential expression patterns in noncoding microRNAs between HESC lines result in distinct differentiation properties [49], indicating that epigenetic phenomena may be regulating these diverse differentiation preferences.

### Conservation of non-CG methylation

Previous studies have shown that the methylation on non-CG sites is widespread in HESCs, but absent in differentiated cells such as fibroblasts. By comparing the genome-wide methylation profiles of three HESC lines, we were able to determine whether these methylated non-CG sites are conserved across different HESC lines. We hypothesized that if they are conserved, they are more likely to be functional, whereas if they are not conserved, they may simply result from higher levels of the DNA methyltransferase DNMT3 in HESCs with respect to differentiated cells, leading to non-specific methylation of non-CpG sites [32].

We observed that the vast majority of non-CG sites are methylated at low levels (that is, less than 30%), indicating that only a small fraction of the cells exhibit methylation at any site within the HESC cell lines. These sites were poorly conserved across the three cell lines, suggesting that they may arise from non-specific activity of methyltransferases. In contrast to these observations, we found that highly methylated (greater then 30%) non-CG sites are strongly conserved between the three lines, and are symmetrically methylated. This suggests that these sites, unlike the lowly methylated ones, may be specifically targeted by DNA methyltransferases. In support of this hypothesis we observed that specific sequence motifs are preferred at these sites, indicating that the higher methylation levels may be driven by sequence specificities of the methyltransferases.

Using our data alone, it is not possible to determine the functional role, if any, of these sites. However, we have found that not only are these highly methylated non-CG sites enriched in splice sites, they are also more methylated than other non-CG sites in genes; they may therefore play a role in regulating transcription in HESCs. Non-CG methylation is found to be more correlated with transcription than CG methylation, and may be preventing spurious transcription initiations [50]. While it is as yet not clear whether the splicing machinery is in any way regulated by the methylation of these sites, it is intriguing that this is yet one more piece of evidence indicating that splicing at chromatin are coupled with DNA methylation in complex ways [22,51,52].

### Allele-specific expression and methylation

Genetic and epigenetic differences between the two parental chromosomes lead to the widespread occurrence of unbalanced transcription of the two alleles. Some studies estimate that as much as one-third of genes (20 to 50%) are transcribed in a significantly unbalanced fashion [13,14,36]. We have developed a novel methodology that exploits genome-wide bisulfite converted DNA sequences to identify locations in the genome that harbor polymorphisms between the two parental chromosomes to identify allele-specific methylation. This methodology allows us to characterize both the genetic and epigenetic differences between the two chromosomes.

We combined the data generated by BS-seq and RNA-seq techniques and developed a novel approach to detect genes with allele-specific expression. Our analysis provides the first genome-wide scan for genes with allele-specific expression that jointly incorporates genome-wide DNA methylation data. Overall, we found that about one-third of all genes show significant allele-specific expression. We determined that about 14% of all CG sites are differentially methylated between the two parental chromosomes. Finally, we found ten genes that are enriched with differentially methylated sites in multiple ESC lines. Six of these genes also have allele-specific expression patterns, suggesting that this imbalance is mediated by allele-specific methylaton. The remaining genes with allele-specific expression were not enriched for differentially methylated CG sites but many of them harbored one or more differentially methylated sites that could be causing the transcriptional imbalance.

Finally, using our approach we are able to 'phase' the methylation patterns of the major and minor alleles (as determined by the RNA-seq data). For the genes that were enriched for allele-specific methylation, we found that one of the two parental chromosomes was completely methylated while the other was unmethylated. These results suggest that our methodology is able to detect genome-wide allele-specific methylation and transcription, as well as phase the methylation pattern of individual genes, thus discovering new genes that are transcriptionally regulated by allele-specific methylation events.

### Methylation of *cis*-regulatory elements

The physical interactions between TFs and their DNA targets have been extensively characterized in many structural studies [53]. It is reasonable to speculate that the methylation status of cytosines in the binding site could significantly affect the binding affinity [42], but this hypothesis has been difficult to test on a genome-wide scale. To address this question, we performed a systematic analysis of the correlation between changes in methylation status at binding sites and the resulting changes in gene expression across the three HESC lines. The expectation was that if TFs are sensitive to the methylation state of their target sequences, then we should observe a significant correlation between this and the resulting gene expression levels.

Using this approach we identified several TFs with significant correlation between the differential methylation in binding sites and their associated expression, suggesting that their binding affinities are affected by the DNA methylation status of the target sequence. We found that most of the methylation-sensitive TFs are more correlated with the methylation levels of the binding sites with expression than neighboring sequences, suggesting that the *cis*-regulatory elements are directly responsible for these effects. The TFs that showed a statistically significant correlation with methylation play important roles in cellular differentiation. We therefore conclude that the methylation state of *cis*-regulatory elements affects transcriptional programs, and the regulation of these sites is critical for the maintenance of pluripotent states.

## Conclusions

We performed a comparative analysis of the genome-wide DNA methylation profiles from three HESC lines. We find that while non-CG sites with low methylation levels are not conserved, heavily methylated non-CG sites are strongly conserved, especially when found within the motif TACAG in splice sites. By combining BS-seq and RNA-seq data we identified a novel set of genes that are likely transcriptionally regulated by methylation in an allele-specific manner. In the analysis of TFBSs, we found several TFs that showed a correlation between methylation and gene expression levels. The correlation between the methylation at binding sites and expression are generally stronger than in the neighboring sequences, suggesting that the methylation state of *cis*-regulatory elements impacts the ability of TFs to bind and regulate transcription.

## Material and methods

### Aligning bisulfite-converted reads

The bisulfite converted reads were aligned against human genome (hg18) using BS Seeker. It converts both the reads and the genome to a three letter alphabet and uses Bowtie [54] to align reads to the reference genome, where up to three mismatches are allowed in our analysis. It is the only aligner that is able to handle reads generated from different library protocols using pre-methylated adapters (H1, H9), or the Dpn1 adapter (HSF1). The pair-end reads from H9 data are mapped as if they were single ended. Finally, BS Seeker post-processes the alignments to remove non-unique and low quality mappings. Reads with more than two methylated non-CG sites in a row were considered non-converted and were discarded. Table S1 in Additional file 1 shows the mapping results. We have less mapped reads in H1 and suspect this could be due to the different mapping criteria and the possible adapter contamination in several read files.

### Extracting conserved differentially methylated regions

To detect genomic regions where one cell line is more methylated than the other, we surveyed all 1-kb windows and calculated the ratio of the methylation levels in the windows between the more methylated cell line and the less methylated one. If the standard Z score of this ratio exceeds two, then this region is considered differentially methylated. The conserved differentially methylated regions are the overlapping differentially methylated regions from all three pairwise comparisons. In our analysis we found 0.11% of the genome is conserved differentially methylated (see Additional file 7 for a list of the conserved differentially methylated regions).

To estimate the FDR of the fraction of the conserved differentially methylated regions, we first randomized the order of the average methylation levels calculated from the genome of each cell line. We then calculated the fraction of the conserved differentially methylated regions in this randomized permutation. The average fraction of the conserved differentially methylated regions from 300 simulations is 0.0006% (standard deviation = 4.7E-7), which gives an estimate of FDR of 0.54%.

### Identifying SNPs

The identification of SNPs was performed in two steps. The first step was to find heterozygous SNPs between two parents. Using BS-seq data, we searched for SNPs to which at least two different alleles are aligned. Specifically, the read coverage at each position has to exceed eight, and the two main alleles cover more than 75% of the reads. The alleles on reads mapped to the negative strand are also included. Since bisulfite sequencing converts unmethylated read C into T on genomic C, read C and T mapped to genomic C on either strand are not included. Finally, the count of allele per genomic position is the average of their read counts from both strands. Between these two alleles, the difference of reads has to be within 20% of their total so the two alleles have close counts of reads.

The second step is to find among these parental SNPs within transcripts the exonic SNPs expressed in only one parental allele. Using RNA-seq data we screened the parental SNPs for those covered by at least four mRNA reads. The allele with more mRNA reads is the major allele and the other the minor allele. The resulting SNPs are the exonic SNPs expressed in only one parental allele. Within the H1 data we found 610,237 (0.02% of genome) heterozygous SNPs, of which 1.6% are exonic SNPs with allele-specific expression.

### Identifying differentially methylated cytosines

Using our list of SNPs, we first separated BS reads mapped to these into two groups based on the two alleles. From the patterns of methylation in these two groups we can reconstruct the methylation state of the two parental chromosomes. For the reads that segregated into two parental groups, we were able to test if the cytosine is differentially methylated between the two parents. Given the probability of observing a methylated read in one parent, which can be estimated from the methylation level from the reads in the parental group, we performed a binomial test to see if the observed methylated reads exceeded expectation. The test was performed twice by switching the parental groups and the larger *P*-value was recorded. We used a 5% FDR to impose a threshold for *P*-values. When cytosines have *P*-values less than this threshold, it implies that the methylation levels between the two parental groups are significantly different.

## Abbreviations

bp: base pair; BS-seq: whole genome bisulfite sequencing; ESC: embryonic stem cells; FDR: false discovery rate; HESC: human embryonic stem cell; HGNC: HUGO Gene Nomenclature Committee; RNA-seq: whole transcriptome shotgun sequencing; SNP: single nucleotide polymorphism; TF: transcription factor; TFBS: transcription factor binding site.

## Authors' contributions

PC, SEJ and MP designed the study. SF performed the experiments. PC, JJ, SEJ and MP analyzed the data. PC and MP wrote the paper. All authors read and approved the final manuscript.

## Supplementary Material

Additional file 1 **Supplementary information**. Includes supplementary Table S1 and Figures S1 to S14. Click here for file

Additional file 2 **Supplementary Table S2**. List of genes associated with differentially methylated CpG islands. Click here for file

Additional file 3 **Supplementary Table S3**. List of the 1,020 genes that are predicted to have allele-specific expression. Click here for file

Additional file 4 **Supplementary Table S4**. List of 75 imprinted genes from the literature. Click here for file

Additional file 5 **Supplementary Table S5**. List of the 110 genes that are enriched with differentially methylated CG sites in at least one cell line. Click here for file

Additional file 6 **Supplementary Table S6**. List of motifs in transcription factors and the correlation coefficients between change of methylation and the associated changes of gene expression at their binding sites, and at the neighboring sequences. Click here for file

Additional file 7 **Supplementary Table S7**. List of differentially methylated regions overlapped across the three HESC lines. Click here for file
