# Supplementary material for: A comparative analysis of DNA methylation across human embryonic stem cell lines
Source: Genome Biol. 2011 Jul 6;12(7):R62. doi: 10.1186/gb-2011-12-7-r62 (PMC3218824; doi:10.1186/gb-2011-12-7-r62)
Supplement: Additional file 1 — Supplementary information. Includes supplementary Table S1 and Figures S1 to S14. [file gb-2011-12-7-r62-S1.DOC]

A comparative analysis of DNA methylation across human embryonic stem cell lines

Pao-Yang Chen, Suhua Feng, Jong Wha Joanne Joo, Steve E. Jacobsen, and Matteo Pellegrini

# Supplementary Information

# Supplementary Tables

### Table S1

Descriptive statistics of the mapping of the three cell lines H1, HSF1, and H9

| Cell line | H1 | HSF1 | H9 (WA09) |
| --- | --- | --- | --- |
| Gender | male | male | Female |
| Data source | Lister et al Nature (2009) | Chodavarapu et al Nature (2010) | Laurent et al Genome Research (2010) |
| Passage | 25, 27 | 49 | 42 |
| Protocol | premethylated adapetr | Cokus et al | premethylated PE adapetr |
| Reads type | Single end | Single end | Paired end |
| Read length | 52~87 | 46,47,50 | 50,75 |
| Reference genome | hg18 | hg18 | hg18 |
| Mapping | BS Seeker, mismatch<=3 | BS Seeker, mismatch<=3 | BS Seeker, mismatch<=3 |
| Number of reads | 1,981,322,270 | 2,093,456,818 | 1,250,691,231 |
| Number of aligned reads | 791,919,144 | 684,155,211 | 792,148,335 |
| % aligned | 40% | 33% | 63% |
| Coverage per strand | 10.43 | 5.22 | 7.54 |
| Covered cytosines | 86% | 70% | 67% |

### Table S2

List of genes associated with differentially methylated CpG islands

Excel file: TableS2.xlsx

### Table S3

List of the 1020 genes that are predicted to have allele-specific expression

Excel file: TableS3.xls

### Table S4

List of 75 imprinted genes from literature

Excel file: TableS4.xls

### Table S5

List of the 110 genes that are enriched with differentially methylated CG sites in at least one cell line

Excel file: TableS5.xls

### Table S6

List of motifs in transcription factors and the correlation coefficients between change of methylation and the associated changes of gene expression at their binding sites, and at the neighboring sequences

Excel file: TableS6.xls

### Table S7

List of differentially methylated regions overlapped across the three hES cell lines

Excel file: TableS7.xls

# Supplementary Figures

##
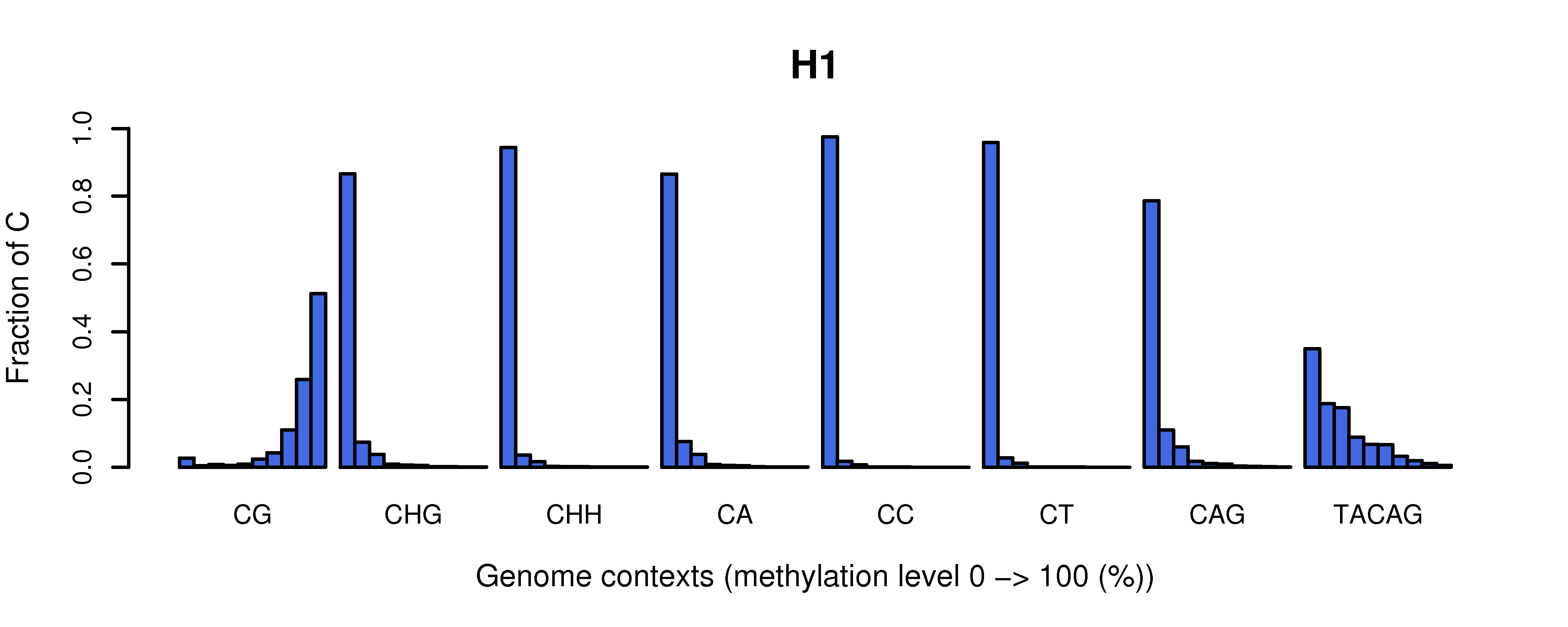

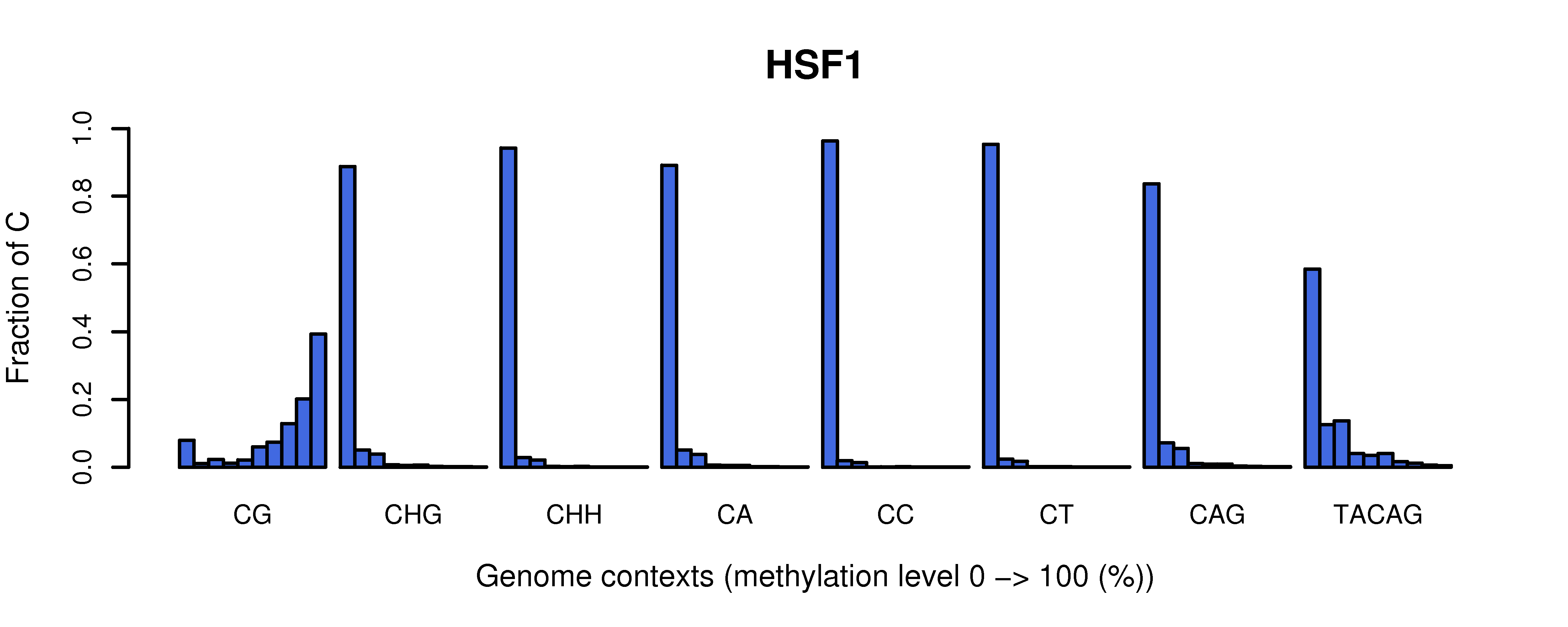


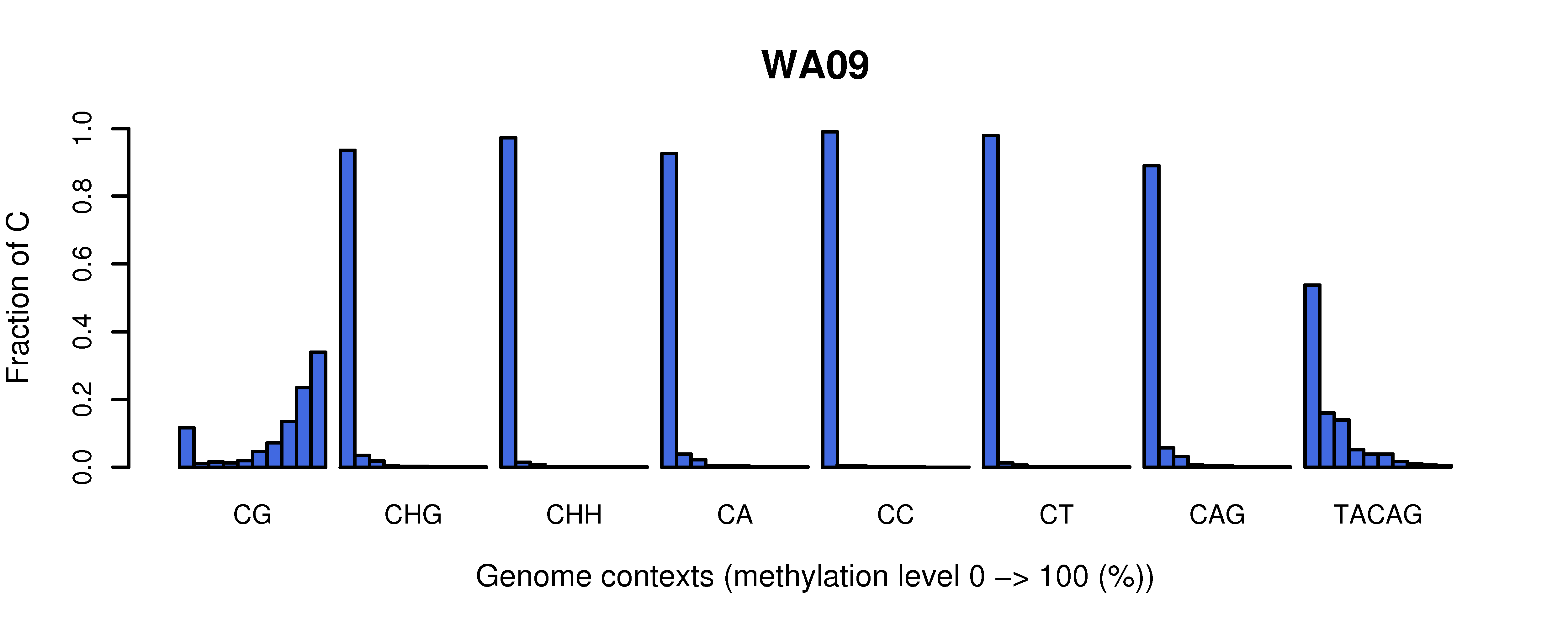


### Figure S1

The distributions of methylation levels in the three HSEC cell lines H1, HSF1, H9

Methylation in cytosines are categorized into CG, CHG, CHH, CA, CC, CT, CAG and TACAG.


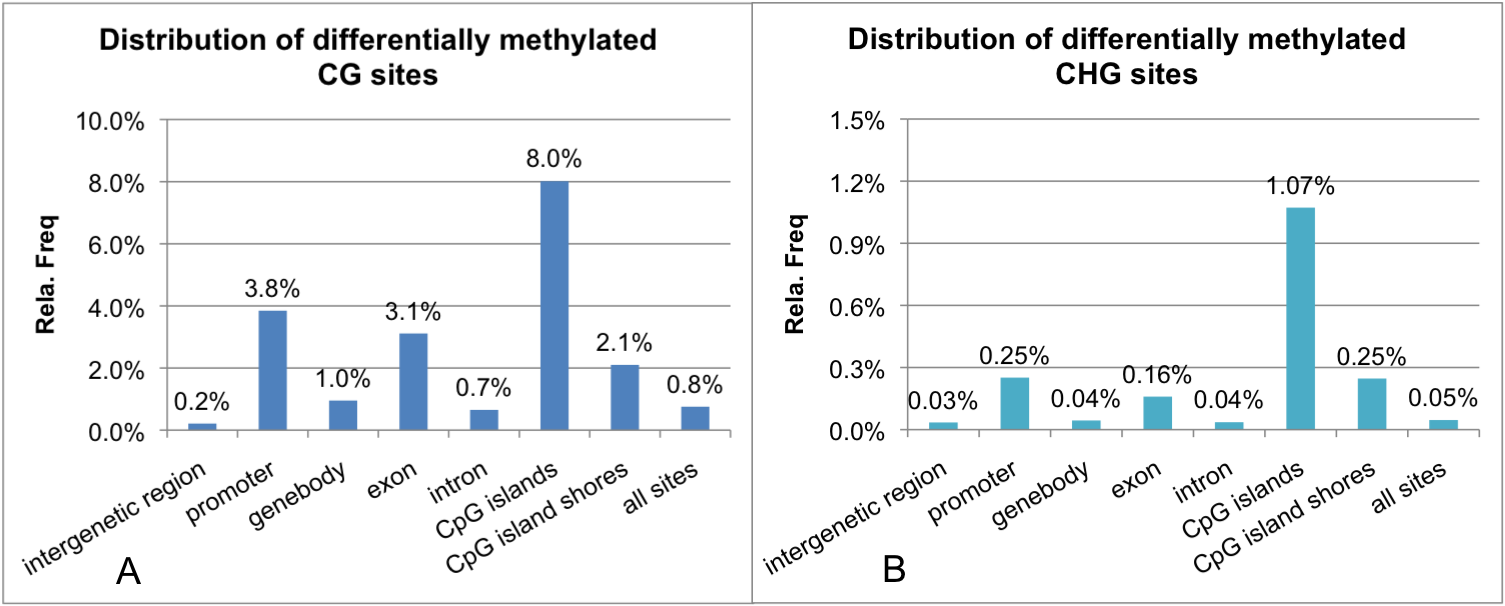


### Figure S2

Distribution of CG (A.) and CHG (B.) from conserved differentially methylated regions.

The relative frequency of genomic feature AAA = (# sites in conserved differentially methylated regions that are in AAA)/(# all sites in conserved differentially methylated regions). Genomic feature AAA can be intergenic region, promoter, genebody, exon, Iintron, CpG islands or CpG island shores.


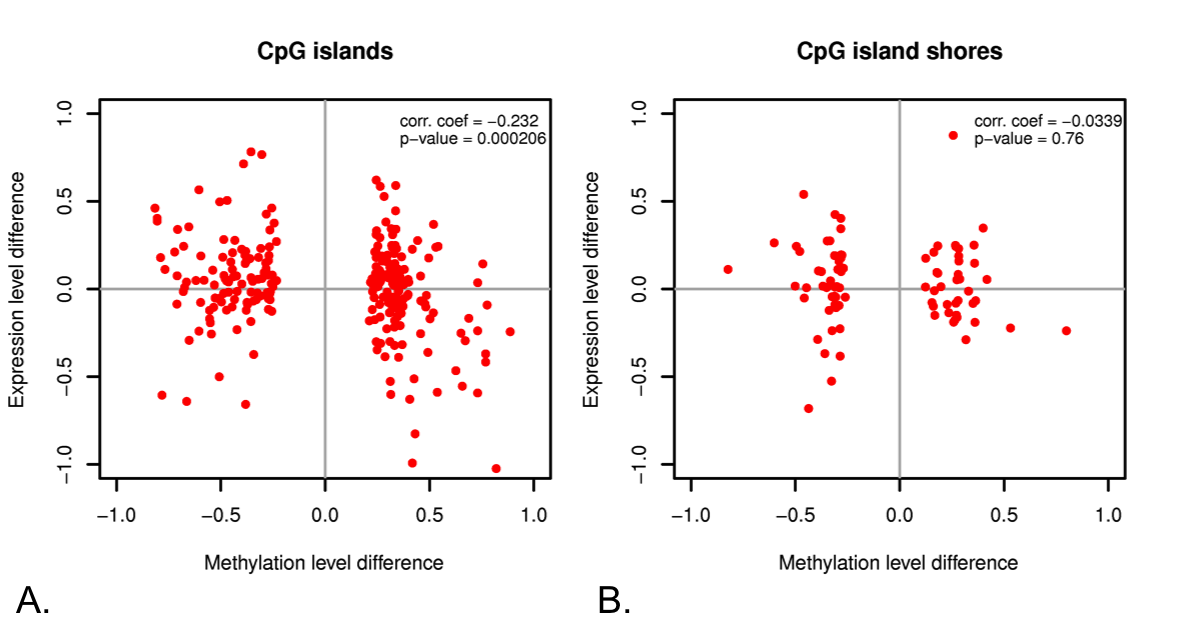


### Figure S3

Scatter plot of differentially methylated CpG islands (A) and CpG island shores (B) associated with their associated differential gene expression levels

###


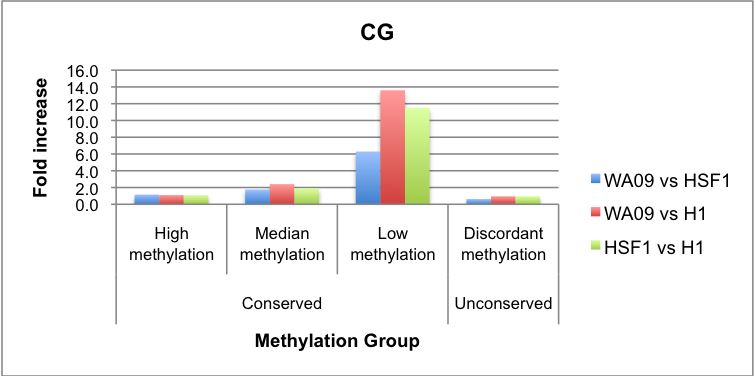

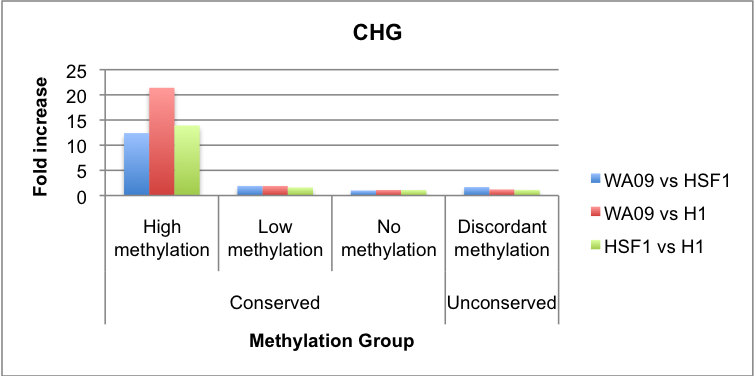


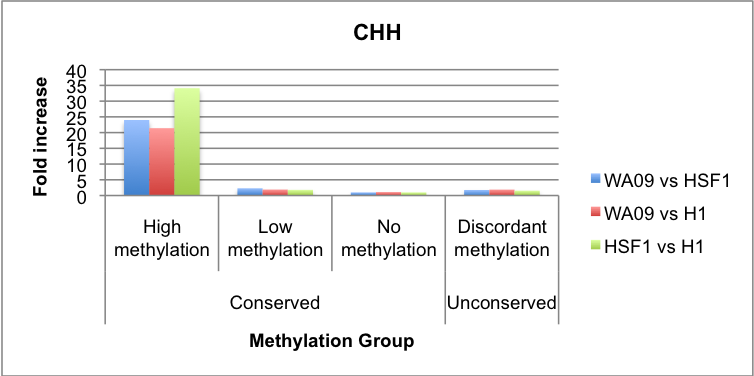


### Figure S4

Fold enrichment of methylation groups from the pairwise comparisons between HESC lines. The sub-figures are methylation in CG (top), CHG (middle), and CHH (bottom).


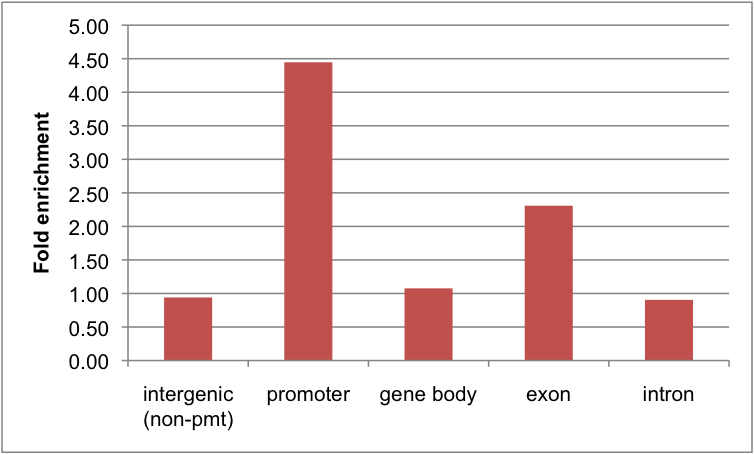


### Figure S5

Fold enrichment of conserved, lowly methylated CG sites (methylation level <33%)


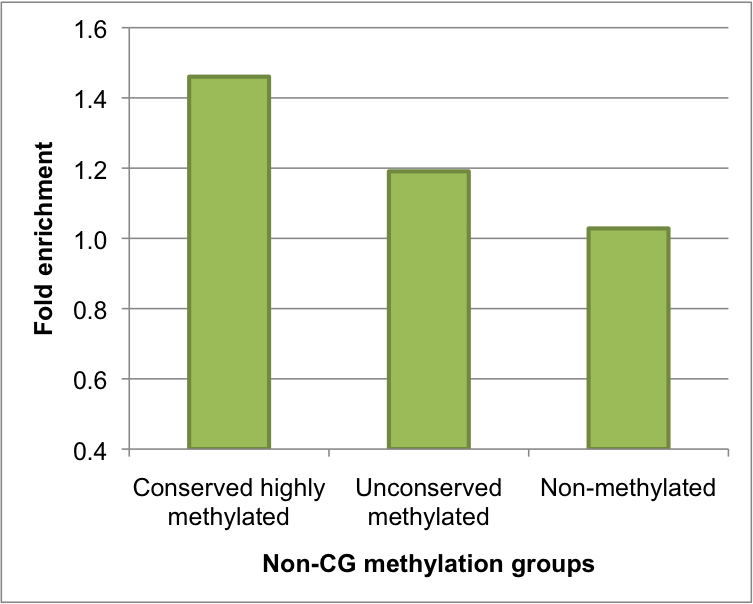


### Figure S6

Fold enrichment of non-CG sites in gene body


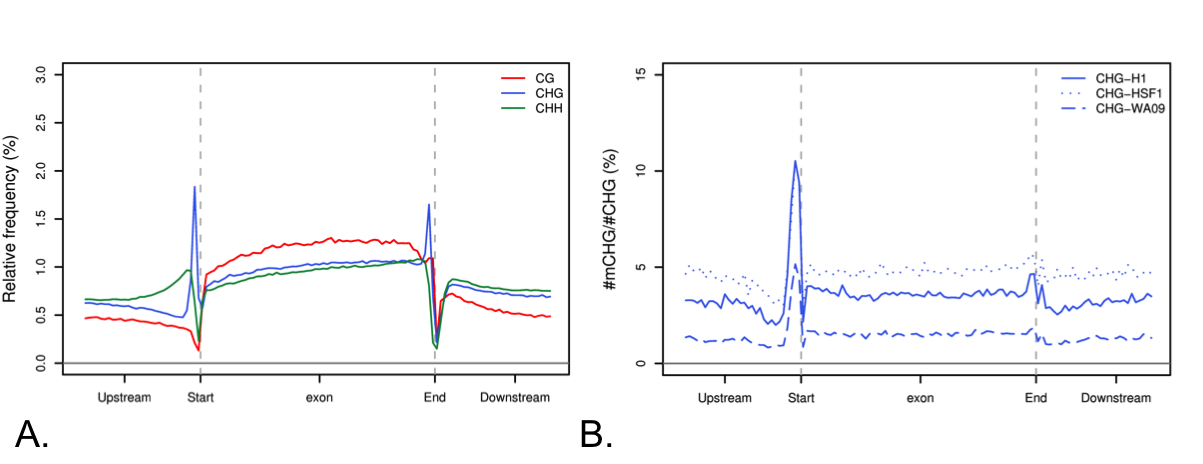


### Figure S7

A. Distribution of CG, CHG and CHH sites in exons. B. Distribution of highly methylated CHG (methylation level >30%) in exons


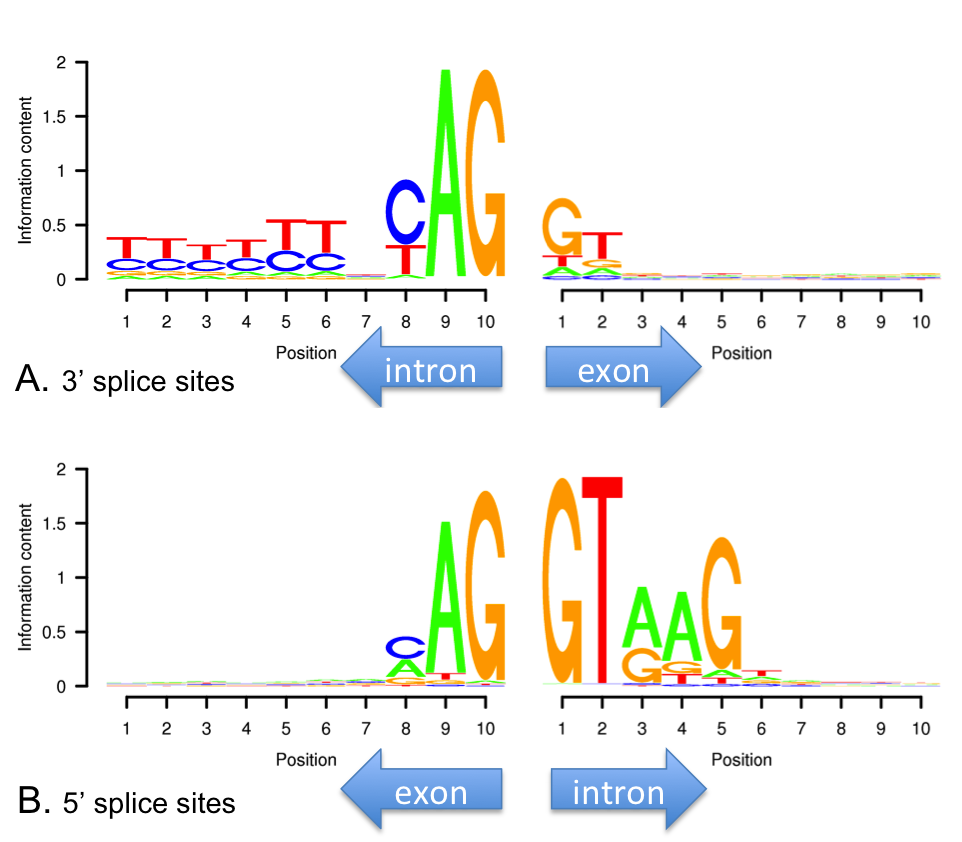


### Figure S8

Sequence motif in (A) 3’ splice sites and (B) 5’ splice sites from upstream 10bp (leftehand) to downstream 10bp (righthand); The X-axis shows the positions listed from 5’ to 3’ end.


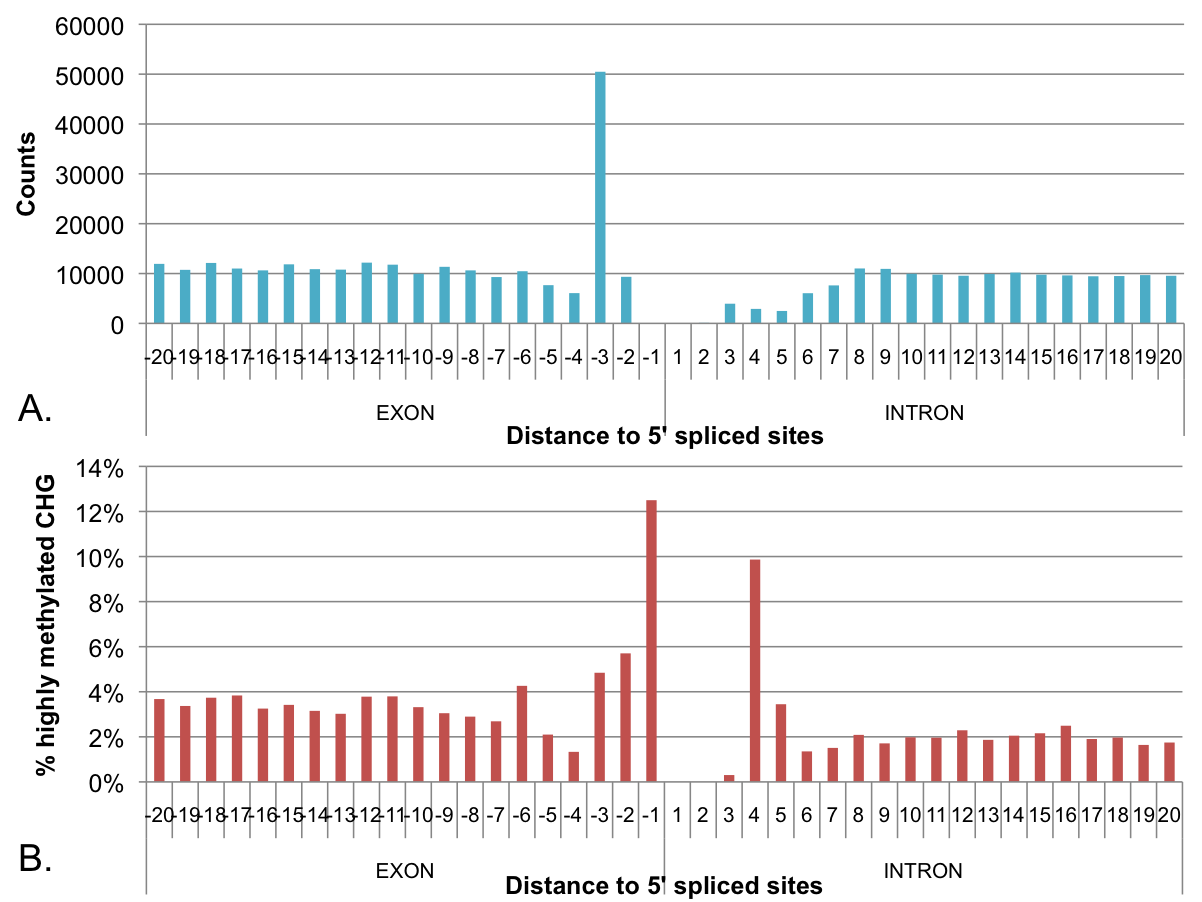


### Figure S9

Counts of CHG sites (A.) and the percent of highly methylated CHG (B.) at each position around 5’ splice sites


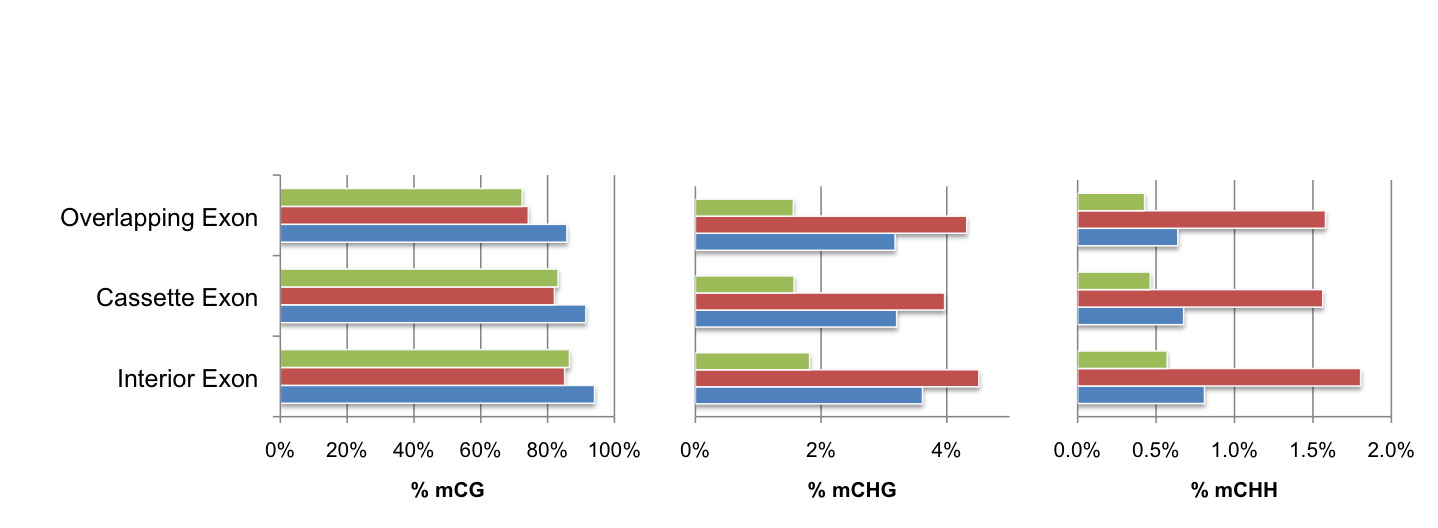


### Figure S10

Methylation level of CG, CHG, and CHH in alternatively spliced exons (cassette exons and overlapping exons) and in interior exons; the result from H9 (WA09) cell lines is in green, HSF1 in red, and H1 in blue. The annotation for cassette exons and overlapping exon are downloaded from UCSC genome browser.


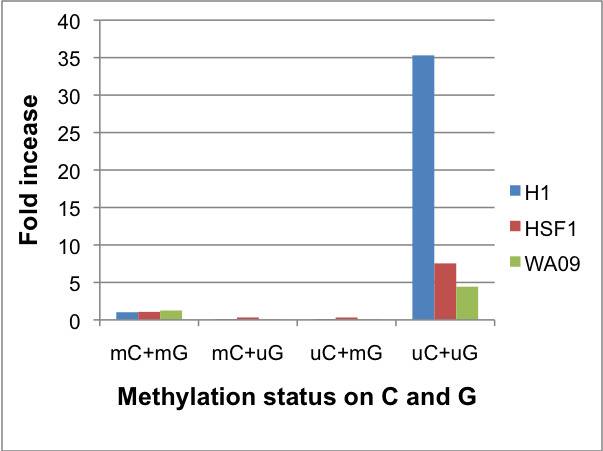


### Figure S11

Fold increase of CG sites by the methylation status of C and G;

CG sites are grouped into 4 groups according to the methylation status of the first C and second G (C on the antisense strand). The methylation status is “m” for methylation level >66% and “u” for methylation level<=33%.


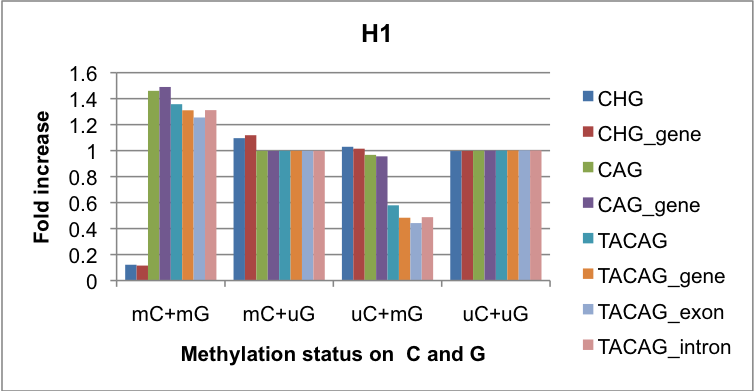


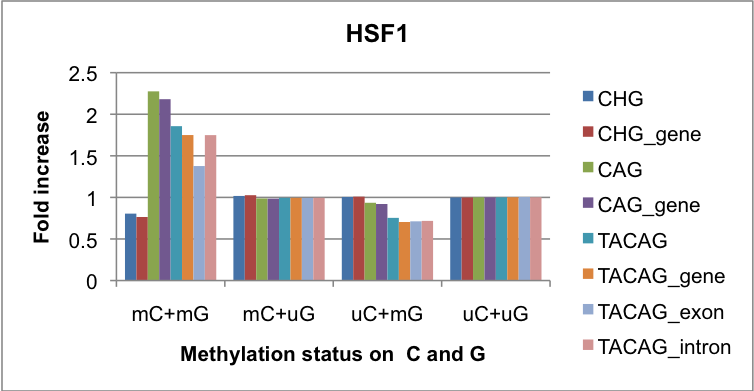


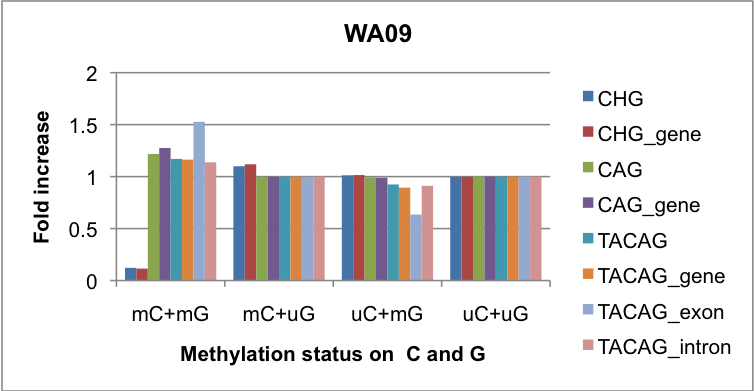


### Figure S12

Fold increase of CHG, CAG and TACAG sites by the methylation status of C and G

In CHG, CAG and TACAG, the sites are grouped into 4 groups according to the methylation status of the first C and second G (C on the complementary strand). The methylation status is “mC” or “mG” for methylation level >30%, and “uC” or “uG” for methylation level=0%. The symmetry of TACAG sites is evaluated on sequence TACAGTA where the first five nucleotides form TACAG on one strand and the last five on the other strand.

In CHG_gene. CAG_gene, TACAG_gene, TACAG_exon, and TACAG_intron, the sites are grouped into 4 groups according to the methylation status of the C on coding strand and second G (C on the antisense strand). The methylation status is “m” for methylation level >30% and “u” for methylation level=0%.


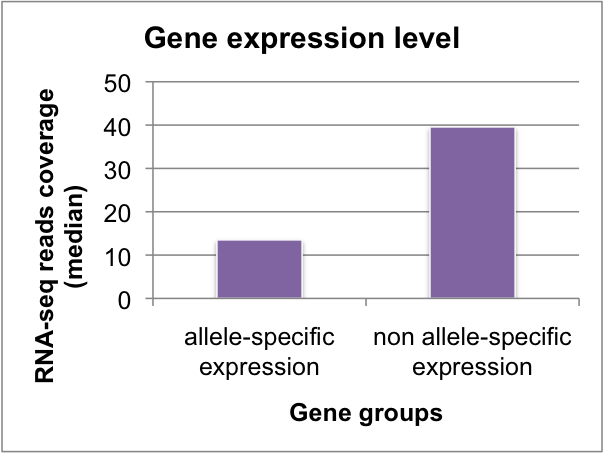


### Figure S13

Gene expression levels in genes with and without allele-specific expression.


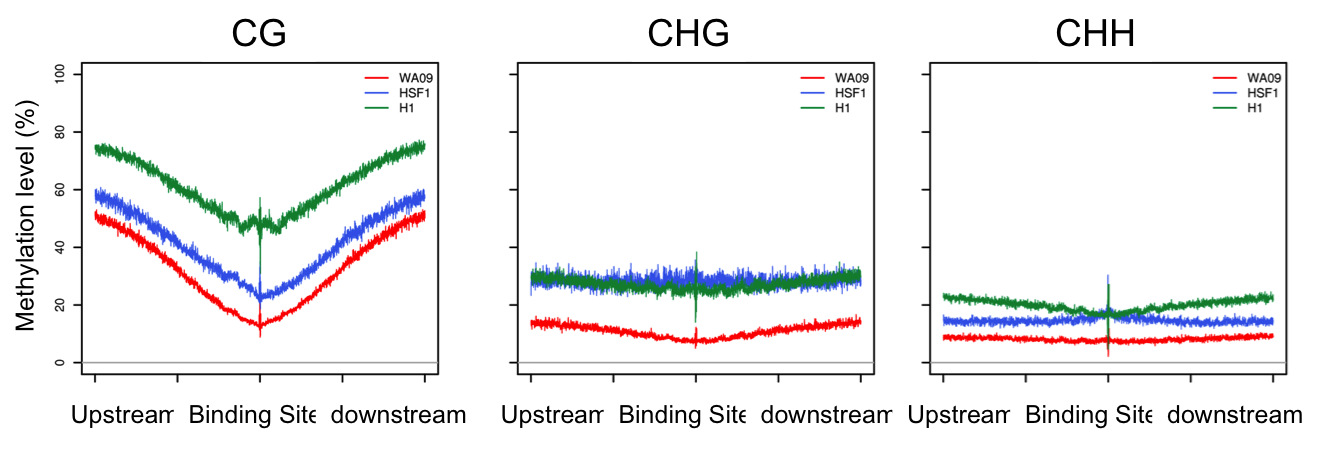


### Figure S14

DNA methylation levels at transcription factor binding sites

The metaplots include upstream 1.5 kb and downstream 1.5kb from the binding sites.
